# Comparison of intraoperative frozen section analysis for sentinel lymph node biopsy during breast cancer surgery for invasive lobular carcinoma and invasive ductal carcinoma

**DOI:** 10.1186/1477-7819-7-34

**Published:** 2009-03-24

**Authors:** James W Horvath, Gary E Barnett, Rafael E Jimenez, Donn C Young, Stephen P Povoski

**Affiliations:** 1 Department of Pathology, The Ohio State University, Columbus, Ohio 43210, USA; 2 Center for Biostatistics, The Ohio State University, Columbus, Ohio 43210, USA; 3 Division of Surgical Oncology, Department of Surgery, Arthur G. James Cancer Hospital and Richard J. Solove Research Institute and Comprehensive Cancer Center, The Ohio State University, Columbus, Ohio 43210, USA

## Abstract

**Background:**

Sentinel lymph node (SLN) biopsy is the standard of care for the surgical assessment of the axilla during breast cancer surgery. However, the diagnostic accuracy of intraoperative frozen section analysis for confirming metastatic involvement of SLNs in cases of invasive lobular carcinoma (ILC) versus that of invasive ductal carcinoma (IDC) has generated controversy secondary to a frequently low-grade cytologic appearance and an often discohesive pattern displayed by metastatic lymph nodes in ILC. In the current report, we present a comparison of intraoperative frozen section analysis for confirming the presence of metastatic disease within SLNs during breast cancer surgery for ILC and IDC.

**Methods:**

We evaluated the results of 131 consecutive cases of ILC from 1997 to 2008 and 133 cases of IDC (selected by a random sequence generator program) from amongst 1163 consecutive cases of IDC from the same time period. All cases had at least one SLN that had both intraoperative frozen section analysis and confirmatory permanent section analysis performed.

**Results:**

No statistically significant difference was found in the sensitivity (67% vs. 75%, P = 0.385), specificity (100% vs. 100%), accuracy (86% vs. 92%, P = 0.172), false negative rate (33% vs. 25%, P = 0.385), negative predictive value (81% vs. 89%, P = 0.158), and positive predictive value (100% vs. 100%) for frozen section analysis for confirming the presence of metastatic disease within SLNs during breast cancer surgery for ILC and IDC.

**Conclusion:**

Since there was no statistically significant difference in sensitivity, specificity, accuracy, false negative rate, negative predictive value, and positive predictive value between frozen section analysis of SLNs for patients with ILC and IDC, the clinical accuracy of confirming metastatic involvement of SLNs on frozen section analysis for ILC should not be considered inferior to the clinical accuracy for IDC. Therefore, frozen section analysis of all SLNs during breast cancer surgery in patients with ILC should remain the standard of care in order to reduce the risk of the need of a later, separate axillary lymph node dissection.

## Background

Sentinel lymph node (SLN) biopsy with intraoperative frozen section analysis has become a standard of care in the surgical staging of the axilla during breast cancer surgery [1-3]. The sensitivity of intraoperative frozen section analysis for identifying nodal metastases within SLNs during breast cancer surgery has been reported to vary widely from the range of 44% to 95% [4-17], with most series reporting the sensitivity of frozen section analysis in the range of 60% to 75% [5,7-9,11-13,15-17].

The difficulty with identifying nodal metastases from invasive lobular carcinoma (ILC) versus invasive ductal carcinoma (IDC) has long been debated within the pathology and surgical communities [17-24]. It has been suggested that individual tumor cells involving the subcapsular sinuses of SLNs in patients with ILC can closely resemble benign lymphocytes and histiocytes when evaluated at frozen section [17,20-24]. Likewise, it has been suggested that the bland cytologic features, round to spindled shape, and discohesive proliferation of ILC cells can make their diagnosis on H&E alone especially difficult [17,20-22,24]. Due to this perceived difficulty in identifying metastatic disease within lymph nodes harvested from patients with ILC, it has been suggested by several authors that false negative frozen section results are more likely in SLN biopsy for ILC as compared to IDC [12,13,17].

The rate of nodal positivity of ILC versus IDC has been extensively compared in the literature [25-36], with most studies showing no significant difference [25,28,30-33,35,36], and only isolated reports showing a significant difference in nodal positivity favoring more in ILC [26] and favoring more in IDC [27,29,34]. Since the diagnostic accuracy of intraoperative frozen section analysis for confirming the presence of metastatic disease within SLNs for ILC versus IDC has long been contended, in the current report, we present a comparison of intraoperative frozen section analysis for confirming the presence of metastatic disease within SLNs during breast cancer surgery for ILC and IDC.

## Methods

### Patient selection

This study was performed under an established Pathology Department protocol approved by Institutional Review Board for the prospectively maintained CoPath database of the Department of Pathology at The Ohio State University.

All female cases of ILC that had undergone frozen section analysis and confirmatory permanent section analysis that was performed on at least one SLN candidate during definitive breast cancer surgery between the time period of 1997 to 2008 were identified from within the CoPath database. This included 131 cases of ILC. From the same time period of 1997 to 2008, all female cases of IDC (n = 1163) that had undergone intraoperative frozen section analysis and confirmatory permanent section analyses that was performed on at least one SLN during definitive breast cancer surgery were also identified from within the CoPath database. Using an internet-available random sequence generator program called "RANDOM.ORG" [37], a similar number of IDC cases (n = 133) were randomly selected from amongst the entire group of IDC cases in order to generate a cohort of IDC cases to be used for direct comparison to the ILC cases.

All female breast cancer cases identified from within the CoPath database that reported mixed lobular/ductal features were excluded from consideration for inclusion in either the ILC group or the IDC group.

### Surgical considerations

The technical details with regards to performing SLN biopsy during breast cancer surgery at The Ohio State University, including the exact methods of injection of radiocolloid and vital blue dye, have been previously described for the time period prior to 2001 [38] and for the time period since 2001 [39].

### Histopathology considerations

At the current time, during intraoperative consultation for frozen section analysis at The Ohio State University, each SLN is grossly sectioned at 0.2 cm interval portions. The most superficial 25% of the thickness of each resulting 0.2 cm SLN tissue section is processed for frozen section analysis, providing at least three separate levels of tissue for frozen section analysis. These frozen sections are then hand-stained by routine Hematoxylin and Eosin (H&E) staining. The remaining tissue of each resulting 0.2 cm SLN tissue section, encompassing 75% of the thickness of that tissue, is then sent for routine processing. Three separate levels (level 1, 2, and 3) on permanent slides are then sectioned at approximately 500 μm intervals and levels 1 and 3 are stained with H&E by an automated staining device, while level 2 is immunohistochemically stained with cytokeratin AE1/AE3. In those specific cases that are reported as having a SLN that is positive for metastatic carcinoma on the frozen section analysis, the level 2 section from each submitted SLN is omitted from undergoing routine cytokeratin AE1/AE3 immunohistochemistry (IHC). We do recognize that the exact methodology of performing frozen section analysis and permanent histopathologic analysis of SLNs for breast cancer cases has changed during the study period from 1997 to 2008.

Metastatic disease within a sentinel lymph node was defined as "macrometastatic" if any given tumor deposit was greater than 2.0 mm and was defined as "micrometastatic" if any given tumor deposit was less than or equal to 2.0 mm. Due to the fact that the study period extends back to 1997 and due to the fact that there was some degree of variability in the reporting style of the multiple original reading pathologists for each of these cases, it was not feasible to accurately further subclassify micrometastatic disease into "micrometastatic" and "submicrometastatic" subclassifications.

It is important to note that the inception of the performance of routine cytokeratin AE1/AE3 IHC for breast cancer cases in which all of the SLNs were reported as negative at the time of the initial frozen section analysis was initiated at The Ohio State University in May 2006. Before May 2006, it was specifically at the discretion of the reading pathologist as to whether or not to utilize cytokeratin AE1/AE3 IHC for further and for more in-depth evaluation of SLNs in any given breast cancer case. Therefore, since it would be difficult to assess the impact of cytokeratin AE1/AE3 IHC on the overall results reported in the current study secondary to the obvious heterogeneity of the application of cytokeratin AE1/AE3 IHC from 1997 through 2006, no attempt was made to differentiate the results of permanent pathologic evaluation based upon whether cytokeratin AE1/AE3 IHC was used or not used.

### Data collection and analyses

Multiple patient variables, primary tumor variables, and SLN variables were evaluated for each case. Data collection of all those variables was simply accomplished by way of retrospective review of the electronic pathology report posted by the original reading pathologist for each case. A re-review of the actual H&E frozen section slides, H&E permanent section slides, and cytokeratin IHC slides for these cases was not undertaken as part of the current analysis. If a given variable was absent from the electronic pathology report posted by the original reading pathologist, that variable was recorded as unknown for that particular case.

The number of true positive (TP), true negative (TN), false negative (FN), and false positive (FP) were determined for frozen section analysis compared to permanent section analysis for the finding of positive SLN for ILC versus IDC. Then, for both ILC and IDC, the sensitivity (TP/(TP+FN)), specificity (TN/(TN+FP)), accuracy ((TP+TN)/total patients), false negative rate (FN/(TP+FN)), negative predictive value (TN/(TN+FN)), and positive predictive value (TP/(TP+FP)) were calculated. All these variables were determined on a per patient basis and were not determined on a per SLN basis.

The software program SPSS 16.0 for Windows (SPSS, Inc., Chicago, Illinois) was used for all statistical analyses. For univariate comparisons of categorical variables, either Pearson chi-square test or Fisher exact test was utilized. Continuous variables were expressed as median (range). For univariate comparisons of continuous variables, one-way analysis of variance (ANOVA) was utilized. All reported univariate P-values were two-sided. All univariate P-values determined to be 0.05 or less were considered to be statistically significant.

## Results

Patient and tumor demographics for ILC and IDC patients are shown in Table 1. ILC patients tended to be older. ILC patients generally had larger tumors and more often displayed multifocal and multicentric disease. ILC generally had a lower histologic tumor grade and were more often estrogen receptor positive, progesterone receptor positive, and Her-2/neu negative. ILC less often had displayed lymphovascular invasion.

**Table 1:** Patient and tumor demographics for invasive lobular carcinomas and invasive ductal carcinomas

	ILC (n = 131)	IDC (n = 133)	Total cases (n = 264)	P-value
Age (years)	59 (35–87)	55 (24–82)	57 (24–87)	0.001
				
Tumor size (cm)	2.0 (0.2–9.0)	1.5 (0.1–6.5)	1.7 (0.1–9.0)	0.006
				
T-stage				
T1	75(58%)	88 (66%)	163 (62%)	0.298
T2	49 (38%)	43 (32%)	92 (35%)	
T3	5 (4%)	2 (2%)	7 (3%)	
T4	1 (1%)	0 (0%)	1 (0.5%)	
				
Tumor focality				
Unifocal	97 (75%)	123 (93%)	220 (84%)	<0.001
Multifocal	19 (15%)	9 (7%)	28 (11%)	
Multicentric	14 (11%)	1 (1%)	15 (6%)	
				
Histologic grade				
Grade 1	40 (31%)	32 (24%)	72 (27%)	<0.001
Grade 2	57 (44%)	52 (39%)	109 (41%)	
Grade 3	17 (13%)	48 (36%)	65 (25%)	
Unknown	17 (13%)	1 (1%)	18 (7%)	
				
ER status				
Positive	121 (93%)	96 (72%)	217 (82)	<0.001
Negative	2 (2%)	29 (22%)	31 (12%)	
Unknown	8 (6%)	8 (6%)	16 (6%)	
				
PR status				
Positive	108 (82%)	81 (61%)	189 (72%)	<0.001
Negative	16 (12%)	44 (33%)	60 (23%)	
Unknown	7 (5%)	8 (6%)	15 (6%)	
				
Her-2/neu				
Positive	13 (10%)	35 (26%)	48 (18%)	0.003
Negative	106 (81%)	87 (65%)	193 (73%)	
Unknown	12 (9%)	11 (8%)	23 (9%)	
				
LVI				
Positive	20 (15%)	41 (31%)	61 (23%)	0.005
Negative	109 (83%)	92 (69%)	201 (76%)	
Unknown	2 (2%)	0 (0%)	2 (1%)	

ILC, invasive lobular carcinoma; IDC, invasive ductal carcinoma; ER, estrogen receptor; PR, progesterone receptor; LVI, lymphovascular invasion

The SLN demographics, including frozen section analysis results, permanent section analysis results, the size of the SLN metastasis, and the classification into macrometastatic disease and micrometastatic disease for ILC and IDC patients are shown in Table 2. No statistically significant difference was noted in any of these SLN demographics variables for ILC versus IDC patients.

**Table 2:** The sentinel lymph node demographics for invasive lobular carcinomas and invasive ductal carcinomas

	ILC	IDC	Total cases	P-value
Frozen section analysis of SLN				
Positive	36 (28%)	33 (25%)	69 (26%)	0.622
Negative	95 (73%)	100 (75%)	195 (74%)	
				
Permanent section analysis of SLN				
Positive	54 (41%)	44 (33%)	98 (37%)	0.171
Negative	77 (59%)	89 (67%)	166 (63%)	
				
Size of SLN metastasis (mm)	6.0 (0.5–25.0)	5.0 (0.1–32.0)	5.5 (0.1–32.0)	0.808
				
Classification of metastatic disease				
Macrometastatic (>2.0 mm)	36 (28%)	28 (21%)	64 (24%)	0.378
Micrometastatic (≤ 2.0 mm)	16 (12%)	12 (9%)	28 (11%)	
Unknown classification	2 (1.5%)	4 (3%)	6 (2%)	
No metastatic disease	77 (59%)	89 (67%)	166 (63%)	

ILC, invasive lobular carcinoma; IDC, invasive ductal carcinoma; SLN, sentinel lymph node

The number of TP, TN, FN, and FP were determined for frozen section analysis compared to permanent section analysis for the finding of a positive SLN for ILC versus IDC patients and are shown in Table 3. The nature of the classification of metastatic disease (i.e., macrometastatic versus micrometastatic) amongst false negative cases for patients with a positive sentinel lymph node for ILC versus IDC is shown in Table 4. No statistically significant difference was noted in any of these variables for ILC versus IDC patients.

**Table 3:** The number of TP, TN, FN, and FP for frozen section analysis compared to permanent section analysis for confirming the presence of metastatic disease within sentinel lymph node candidates for invasive lobular carcinomas and invasive ductal carcinomas

	ILC	IDC	Total cases	P-value
TP	36	33	69	0.622
TN	77	89	166	0.171
FN	18	11	29	0.155
FP	0	0	0	------

Total	131	133	264	

ILC, invasive lobular carcinoma; IDC, invasive ductal carcinoma; TP, true positive; TN, true negative; FP, false positive; FN, false negative

**Table 4:** The nature of the classification of metastatic disease amongst false negative cases for patients with a positive sentinel lymph node for invasive lobular carcinomas and invasive ductal carcinomas

False negative cases	ILC	IDC	Total cases	P-value
Macrometastatic (>2.0 mm)	5 (28%)	2 (18%)	7 (24%)	0.807
Micrometastatic (≤ 2.0 mm)	11 (61%)	8 (73%)	19 (66%)	
Unknown classification	2 (11%)	1 (9%)	3 (10%)	

Total	18 (100%)	11 (100%)	29 (100%)	

ILC, invasive lobular carcinoma; IDC, invasive ductal carcinoma

The sensitivity, specificity, accuracy, false negative rate, negative predictive value, and positive predictive value of frozen section analysis compared to permanent section analysis for the finding of positive SLN for ILC versus IDC patients were calculated and are shown in Table 5. No statistically significant difference was noted in any of these variables for ILC versus IDC patients.

**Table 5:** The sensitivity, specificity, accuracy, false negative rate, negative predictive value, and positive predictive value of frozen section analysis compared to permanent section analysis for the finding of a positive sentinel lymph node for invasive lobular carcinomas and invasive ductal carcinomas

	ILC	IDC	Total cases	P-value
Sensitivity	67%	75%	70%	0.385
Specificity	100%	100%	100%	------
Accuracy	86%	92%	89%	0.172
False negative rate	33%	25%	30%	0.385
Negative predictive value	81%	89%	85%	0.158
Positive predictive value	100%	100%	100%	------

ILC, invasive lobular carcinoma; IDC, invasive ductal carcinoma

## Discussion

The primary reason for undertaking this current analysis was the fact that it has been the longstanding general opinion of many surgical pathologists within the pathology community, including our own, that SLNs in ILC cases are notoriously more difficult to interpret, especially at the time of frozen section analysis. This longstanding contention has been eloquently addressed and debated within the literature [18-24]. Best articulated by Creager et al [21], although not necessarily agreed upon by their group, this longstanding contention specifically asserts that the intraoperative detection of ILC can be highly problematic secondary to its low-grade cytomorphology and its tendency to infiltrate metastatic sites in a single cell pattern. This assertion that was articulated by Creager et al [21] suggests that such architectural and cytomorphologic features of ILC within a given metastatic lymph node can result in occasionally missing even relatively large nodal metastases on intraoperative frozen section evaluation that are then only discovered, much to the surprise of the pathologist and surgeon, on permanent H&E sections and/or cytokeratin AE1/AE3 IHC stained sections. In this regard, our goal was to compare the diagnostic accuracy of intraoperative frozen section analysis for confirming the presence of metastatic disease within SLNs during breast cancer surgery for ILC and IDC, in order to confirm or dispel the above, longstanding contention.

In our study, the sensitivity of frozen section analysis (67% for ILC patients, 75% for IDC patients, and 70% for all patients) was well within the range of sensitivity for frozen section analysis results (i.e. 60% to 75% range) in most previously reported series in the literature for SLN biopsy during breast cancer surgery [5,7-9,11-13,15-17]. Therefore, our frozen section analysis results, based on sensitivity, are highly consistent with the mainstream practice of intraoperative frozen section analysis for SLN biopsy during breast cancer surgery.

Likewise, in our study, we did not find a statistically significant difference in the false negative rate for frozen section analysis for SLN biopsy for ILC as compared to IDC (33% for ILC, 25% for IDC, P = 0.385). Although this may initially seem surprising to some, the vast majority of the literature supports the routine use of intraoperative frozen section analysis for SLN biopsy during breast cancer surgery for ILC cases [10,12,13,16,21,24,40,41]. Nevertheless, several authors have previously reported that false negative frozen section results are more likely in SLN biopsy for ILC as compared to for IDC [12,13,17].

Leidenius et al [12] analyzed a total of 375 breast cancers and reported that the false-negative rate for frozen section analysis during SLN biopsy was more common for ILC than IDC (28% versus 8%, P < 0.01) in an overall analysis of 102 ILC versus 194 IDC. In our estimation, the distribution of tumor types (i.e., ILC versus IDC) reported by Leidenius et al [12] is very perplexing. In their series [12], they reported seeing 102 cases of ILC among a total of 375 total breast cancer cases during a 22 month time period from 01/02/2001 to 11/7/2002 in Helsinki, Finland. This signifies that ILC makes up an astonishing 27.2% of all the breast cancers seen in Helsinki, Finland. This is in stark contrast to the maximum of 10% to 15% of ILC cases that are generally seen among all presenting breast cancers within the United States [17,26,34,35] and worldwide [27,30,31,33,36,41,42]. Secondly, they found an unusually low false negative rate of frozen section analysis for SLNs for IDC cases (8%) as compared to for ILC cases (28%) [12]. In contrast, most series in the literature generally report a false negative rate of frozen section analysis for SLN biopsy for breast cancer cases is in the range of anywhere from 26% to 56% [5,7,8,10,11,13-15,17], including our own current series in which the false negative rate of frozen section analysis for SLN biopsy was 33% for ILC, 25% for IDC, and 30% for all breast cancer cases. This particular aspect of Leidenius et al [12] reported series can not be easily explained in view of the rest of the reported literature and casts some doubt into their results and contention that false negative frozen section results are more likely in SLN biopsy for ILC as compared to for IDC.

Similarly, Holck et al [13] analyzed a total of 265 breast cancers and reported that false negative findings were overrepresented for ILC on frozen section analysis during SLN biopsy (i.e., 5 of 28 or 17.9% of the false negative frozen section results were from ILC). Despite the fact that Holck et al [13] made this statement, they failed to specify within their paper exactly how many ILC cases they analyzed from among the 265 breast cancers they saw in Hilleroed, Denmark over a 20 month period of time from February 2001 through September 2002 and did not provide enough raw data or P-values to verify their claim for overrepresentation.

Lastly, Chan et al [17] most recently analyzed a total of 5298 breast cancers and reported that the false negative rate for frozen section analysis during SLN biopsy was more common for ILC than IDC (47.6% versus 37.8%, P = 0.006) in an overall analysis of 574 ILC versus 4531 IDC. Despite the statistically significant difference between ILC and IDC that they reported, Chan et al [17] went on to state in their discussion that "although this difference is statistically significant, it may not be clinically significant, as frozen section successfully detected a majority of SLN metastases in both groups". Likewise, Chan et al [17] never concluded in their report that frozen section analysis of SLNs during breast cancer surgery for ILC should be abandoned.

Therefore, it is reasonable to conclude, based on our results showing no significant statistical difference in the false negative rate on frozen section analysis for SLNs in ILC versus IDC cases, that intraoperative frozen section analysis of SLNs during breast cancer surgery for ILC should remain an important standard of care. This allows for accurate intraoperative assessment of the nodal status of the axilla, thus allowing the surgeon to appropriately proceed with an immediate concomitant axillary lymph node dissection based upon the intraoperative finding of a positive SLN and thus minimizing the need for an additional, subsequent, delayed axillary procedure. Clearly, intraoperative frozen section analysis during SLN biopsy is no less important for ILC than it is for IDC.

Despite the fact that our results do not show any significant difference in the diagnostic accuracy of intraoperative frozen section analysis using hand-stained, routine H&E staining for confirming the presence of metastatic disease within SLN candidates during breast cancer surgery for ILC and IDC, several relevant issues with regards to the potential impact of IHC on the detection rate of axillary lymph node metastases in cases of ILC seem to be worth further discussion.

Recently, Tan et al [43] retrospectively analyzed a cohort of 368 previously presumed node-negative breast cancer patients (319 with IDC and 49 with ILC) that were treated with axillary lymph node dissection between 1976 and 1978 and who had 20-year follow up. They retrospectively performed IHC in order to attempt to identify occult axillary lymph node metastases based upon IHC detection versus historical standard H&E detection. From their retrospective performance of IHC, they were able to identify three very important pathological features that were specifically attributable to ILC cases. First, ILC cases had a higher rate of conversion from node negative to node positive than did IDC cases (40% versus 20%). Second, ILC cases had an over-representation IHC-detected disease versus H&E-detected disease (36% versus 15%). Third, ILC cases had an over-representation among patients with single-cell metastases versus clustered metastases (59% versus 7%). Certainly, these pathological features demonstrate the potential impact that IHC may have on the overall diagnostic accuracy of confirming the presence of metastatic disease within SLN candidates for ILC cases. Nevertheless, since this study cohort [43] represents a group of patients treated in the pre-SLN biopsy era, these IHC results have no direct correlation to or bearing upon the current intraoperative assessment of frozen section analysis for confirming the presence of metastatic disease within SLN candidates during breast cancer surgery for ILC.

More relevant to the SLN biopsy era, Patil and Susnik [24] recently retrospectively reviewed 76 patients with ILC undergoing SLN biopsy during the time period of 2003 to 2007. Of the 76 cases, 24 cases (32%) were positive for metastatic disease (21 macrometastatic and three micrometastatic), and 14 cases (18%) demonstrated isolated tumor cells (submicrometastatic) on IHC only. All macrometastatic cases (n = 21) and two of three micrometastatic cases were identified on standard H&E evaluation alone. All cases of isolated tumor cells (n = 14) and one micrometastatic case were detected on IHC alone. Therefore, based on IHC, they officially changed the axillary lymph node status from negative to positive in only one case of micrometastatic disease. They concluded that upstaging very rarely occurred with the use of IHC [24]. Likewise, they concluded that yielding a diagnosis of isolated tumor cells, which prognostically is not completely understood at this time, rarely results in any deviation of the treatment plan and provides no additional advantage over that of a thorough standard H&E evaluation [24].

A last relevant point of discussion with regards to IHC is that several groups have advocated the specific use of rapid intraoperative IHC in addition to frozen section H&E stained levels and possibly touch imprints cytology. Leikola et al [23] analyzed 995 breast cancer patients (523 with IDC and 245 with ILC) undergoing SLN biopsy during the time period of 2001 to 2007. They demonstrated that rapid intraoperative IHC on frozen sections analysis improved the sensitivity of detecting metastatic disease within SLNs from 66% (without IHC) to 87% (with IHC) for patients with ILC (P = 0.02). Similarly, Weinberg et al [22] analyzed 59 breast cancer patients with ILC using rapid intraoperative IHC on touch imprint cytology. They demonstrated that their sensitivity for identifying a SLN containing metastatic disease was increased from 41.9% (without IHC) to 54.8% (with IHC) using rapid intraoperative IHC on touch imprint cytology and concluded that rapid intraoperative IHC on touch imprint cytology enhances the intraoperative diagnosis of SLN metastases in patients with ILC. However, no specific P-values were reported by Weinberg et al [22] to support their data or their conclusions.

While IHC is currently widely utilized at many institutions around the globe as part of standard histopathologic evaluation of SLNs for breast cancer, the specific relevance and impact of IHC can not be directly addressed within the context of the findings of our current report, since we did not specifically analyze IHC findings as an independent variable within our overall assessment of the diagnostic accuracy of intraoperative frozen section analysis for confirming the presence of metastatic disease within SLNs candidates during breast cancer surgery for ILC and IDC. Obviously, the specific impact of IHC on the overall assessment of the diagnostic accuracy of intraoperative frozen section analysis for confirming the presence of metastatic disease is multifactorial and is beyond the scope of our current discussion.

## Conclusion

Since there was no statistically significant difference in sensitivity, specificity, accuracy, false negative rate, negative predictive value, and positive predictive value between frozen section analysis of SLNs for patients with ILC and IDC, the clinical accuracy of confirming metastatic involvement of SLNs on frozen section analysis for ILC should not be considered inferior to the clinical accuracy for IDC. Therefore, frozen section analysis of all SLNs during breast cancer surgery in patients with ILC should remain the standard of care in order to reduce the risk of the need of a later, separate axillary lymph node dissection.

## Abbreviations

SLN: sentinel lymph node; ILC: invasive lobular carcinoma; IDC: invasive ductal carcinoma; IHC: immunohistochemistry; ER: estrogen receptor; PR: progesterone receptor; LVI: lymphovascular invasion; TP: true positive; TN: true negative; FN: false negative; FP: false positive

## Competing interests

The authors declare that they have no competing interests.

## Authors' contributions

JWH was involved in the study design, data collection, and writing and editing all aspects of this manuscript. GEB and REJ were involved in the study design and editing this manuscript. DCY was involved in the study design, data analysis, and editing this manuscript. SPP was involved in the study design, data analysis, writing and editing all aspects of this manuscript, and represented the senior physician overseeing the project. All of the authors have read and approved the final version of this manuscript.
